# Genetic Confirmation of Clonal Spread of *Candida auris* from Southern to Northern Nevada

**DOI:** 10.3390/jof11060445

**Published:** 2025-06-12

**Authors:** Paul J. Resong, Joseph Lee, Adam Vazquez, David Hess, Kirk Bronander, Samuel A. Lee

**Affiliations:** 1 Department of Medicine, University of Nevada Reno School of Medicine, Reno, NV 89250, USAjosephrlee@med.unr.edu (J.L.);; 2 Nevada State Public Health Laboratory, Reno, NV 89503, USA

**Keywords:** *Candida auris*, case report, geographic spread, Las Vegas, outbreak, Northern Nevada

## Abstract

*Candida auris* is an emerging fungal pathogen characterized by high levels of antifungal drug resistance and hospital outbreaks in a global distribution. Since introduction to the United States, it has been identified most frequently in New York, Illinois, California, Florida, and Nevada. Its surge poses significant risk as a nosocomial infection with multi-drug resistance, with clades bearing resistance to fluconazole, micafungin, and amphotericin B. Within the state of Nevada, and specifically the greater Las Vegas area in the southern part of the state, there are ongoing outbreaks from clade I and clade III, with 1728 confirmed clinical cases identified as of January 2025. In northern Nevada, three clinical cases have been identified to date, with two occurring at our facility. Both patients had been hospitalized at facilities in Las Vegas, Nevada. The *C. auris* strains isolated from these two cases have been identified as belonging to clade III and demonstrate resistance to fluconazole. Genome sequencing of the *C. auris* isolates indicates close genetic identity to strains from the Las Vegas outbreak. These data indicate that the spread of these clonal isolates is due to hospitalization and subsequent patient relocation to northern Nevada, revealing the ongoing importance of screening for geographic spread.

## 1. Introduction

*Candida auris* is a drug-resistant opportunistic yeast which was first isolated in 2009 in Japan and has since rapidly spread globally [1]. A rapid acceleration of hospital-acquired *C. auris* cases in the United States was identified during the coronavirus (COVID-19) pandemic, likely as a result of increased hospitalizations nationwide [2]. There are six identified clades, with variation in antifungal resistance within each clade [3,4,5]. In 2016, several clades were initially identified via matrix-assisted laser desorption ionization time-of-flight mass spectrometry (MALDI) [6]. Since then, identification of *C. auris* clades has favored both geographic and genomic sequencing approaches, as high levels of single nucleotide polymorphisms (SNP) are noted between clades. Specifically, different clades will show SNP differences in the tens of thousands, while SNP differences within clades are typically less than one hundred. The distribution of these clades are as follows: clade I (south Asia), clade II (east Asia), clade III (Africa), clade IV (South America), clade V (Iran), and clade VI (Indomalayan) [3,4,5].

Concerningly, there is a high mortality rate associated with *C. auris* after fungemia and dissemination occurs, and treatment may be complicated by inherent resistance to fluconazole, multi-drug resistance, or extensive drug resistance [7]. Additionally, with the ability to form biofilms, and resistance to several common antiseptics used in healthcare facilities [8], nosocomial infection poses a significant risk to vulnerable patients. Both residents of long-term care facilities, such as skilled nursing facilities (SNF) and patients who are hospitalized for long periods of time, have been identified as having a higher risk of *C. auris* infection [9,10].

Amongst the clades, antifungal resistance varies and depends strongly on how closely related the clades are to each other. While there are no established MIC (minimum inhibitory concentration) breakpoints for *C. auris* yet, the Centers for Disease Control has published tentative breakpoints based on extrapolation from other *Candida* species and expert opinion. Clades I and III show the highest resistance rates (94–96% resistant to fluconazole) [11]. Clade IV shows resistance to azole and echinocandin antifungals. However, clades II, V, and VI demonstrate lower resistance rates to all antifungals, including fluconazole [11].

Within the state of Nevada, cases have primarily been reported in southern Nevada, with Las Vegas having an ongoing outbreak which began in August of 2021 [12]. As of 13 January 2025, with unpublished Nevada State Public Laboratory data available upon public record request, there have been a total of 5118 clinical isolates noted in the state of Nevada, comprising 3390 cases of colonization and 1728 clinical cases. Ongoing epidemiological investigation has shown this to be a dual clades I and III outbreak [12]. From this outbreak, three distinct lineages have been detected from clade I, and one distinct lineage from clade III [13]. Additionally, *C. auris* has been isolated from sources including urine, blood, wound, axillary and groin cultures during the course of this ongoing outbreak [12]. All clinical isolates in Nevada are stored in the Nevada BioProject (PRJNA846332). However, in northern Nevada, only three clinical cases have been reported since 2018.

Herein, we report the clinical features of two cases of *C. auris* infections in northern Nevada, with both having recent hospitalization in southern Nevada, and the results of genome sequencing of the clinical isolates to determine the originating source of infection.

## 2. Materials and Methods

The following methods were used by the Nevada State Public Health Laboratory to identify the lineage of *C. auris* samples.

### 2.1. C. auris Collection

Clinical *C. auris* isolates were received from submitting institutions on Sabouraund Dextrose agar slants (Hardy Diagnostics, Santa Maria, CA USA). These samples were streaked for single colonies on HardyCHROM™ *Candida* agar plates (Hardy Diagnostics, Santa Maria, CA, USA) and incubated a temperature of 36 C for 2–5 days. Single colonies were sub-cultured on HardyCHROM™ *Candida* agar plates at 36 C for 2–5 days before DNA extraction.

### 2.2. Antimicrobial Susceptibility Testing

*C. auris* antimicrobial susceptibility testing (AST) was performed using microbroth dilution and predefined gradient of antibiotic concentrations (Etest) methods. A patient isolate was grown on a SabDex agar plate (S2 Media, Spokane Valley, WA, USA), incubated at 30 °C in ambient air for 24 h, and used to make 0.5 McFarland inoculum (Hardy Diagnostics, Santa Maria, CA, USA) suspension in demineralized sterile water. The 0.5 McFarland suspension was measured by spectrophotometer to verify the 0.5 McFarland (80–82% transmittance). Twenty microliters of the 0.5 McFarland suspension were added into 11 mL of RPMI broth tube (S2 Media, Spokane Valley, WA, USA) and 100 μL of the RPMI diluted sample was distributed into each well of a 96-well plate pre-loaded with antibiotics. Plates were then incubated along with control plates for 24 h at 35 °C. The same 0.5 McFarland inoculum suspension was used to inoculate an RPMI agar plate using a sterile cotton swab. A single Amphotericin B Etest strip (bioMérieux, Marcy-l'Étoile, France) was applied to the middle of the agar surface using sterile forceps and incubated along with control plates for 24 h at 35 °C. The AST of the microbroth dilution panel was read using a parabolic magnifying mirror to determine the MIC (lowest concentration where there is ≤50% growth compared to the growth control well). For the Amphotericin B Etest, MIC was interpreted at a value where there is 100% growth inhibition (number above where the ellipse intercepts Etest strip).

### 2.3. Whole Genome Sequencing of C. auris

Genomic DNA used for sequencing was extracted using a combination of bead-beating (FastPrep-24, MP Biomedicals, Irvine, CA, USA) and magnetic-bead purification (Maxwell RSC 48, Promega, Madison, WI, USA). First, isolates from Sabouraud Dextrose were added to agar plates and mixed with silica beads (Lysing Matrix C, MP Biomedical). Cells were mechanically sheared with 2 cycles at 6.0 m/s for 30 s, with a 5-minute pause between (FastPrep-24, MP Biomedical). Genomic DNA was isolated using the PureFood Pathogen Kit (Promega) on a Maxwell RSC 48 (Promega), following the manufacturer’s protocol. Genomic DNA was library prepped using the DNA Prep Kit (Illumina, San Diego, CA, USA), following the manufacturer’s recommended protocol to use a STARlet automated liquid handler (Hamilton Company, Reno, NV, USA). Paired-end sequencing (2 × 151) was performed using Illumina’s MiniSeq and NovaSeq 6000 to a minimum depth of 35× average coverage.

### 2.4. TheiaEuk Implementation

The TheiaEuk_Illumina_PE_PHB (v3.0.1) workflow performed the assembly, quality assessment, and genomic characterization of fungal genomes (described in Ambrosio et al., 2023) [14]. This cloud-native workflow was implemented in the Workflow Description Language and was operationalized on the Terra.bio platform (https://terra.bio/, accessed on 2 January 2025). Input reads were preprocessed with a raw-read quality assessment followed by read cleaning (quality trimming and adapter removal), and then an additional quality assessment of the cleaned reads. Subsequently, de novo assembly was performed using the Shovill package (1.1.0) with SKESA set as the default assembler. Once the assembly had been generated, an assembly quality assessment was performed using QUAST (5.0.2). Using the assembly, species taxon identification was performed by GAMBIT (1.0.0) [15]. For some taxa identified, taxa-specific sub-workflows were automatically activated, launching additional taxa-specific characterization tools, including a GAMBIT-based clade-typing tool (v0.5.1) and antifungal resistance detection performed using Snippy (v4.6.0) variant calling with a custom query for genes known to carry antifungal-resistance conferring mutations.

### 2.5. C. auris Specific Subroutines Within TheiaEuk

Upon the taxonomic assignment of *C. auris* to a sample, TheiaEuk_Illumina_PE_PHB automatically triggered two taxa-specific sub-workflows [14]. First, a clade-typing workflow was launched. Clade-typing was performed using a modified version of the GAMBIT module to determine which of the five clade-specific references most closely matched the query sequence. The output of the clade-typing module included the clade assignment as well as a clade-specific annotated genome, which was then passed to the antifungal resistance detection module. Second, Snippywas used to align reads to the annotated reference genome and call variants in *FKS1*, *ERG11* and *FUR1*. The variants were annotated with the genes in which they were found because the input reference genome was annotated. The variants were then queried for any that occur within genes known to contain resistance conferring mutations. This method was used rather than reporting only known resistance conferring mutations to ensure that novel resistance conferring mutations were not ignored.

### 2.6. Shared SNP Analysis

Shared SNP analysis was performed as described in [13]. Briefly, this analysis used the vcf file from kSNP3 (v.3.3.1) which displayed whether each SNP was present, absent, or unassembled for each input genomes. This analysis focused only on SNPs that were assembled in each input genome and then filtered out from that group, SNPs that were in every input genome (save the reference genome—GenBank Accession GCA_002775015.1), and SNPs that were unique to only one of the input genomes. The SNPs that remain were referred to as “shared SNPs”, falling somewhere between unique and present in all genomes in the query set. These SNPs were manually clustered to form groups that had unique patterns of shared SNPs.

## 3. Cases

### 3.1. Case 1

A 61-year-old male resident of a skilled nursing facility (SNF) presented in August 2023 for surgical debridement of a stage IV sacral decubitus ulcer extending to bone. The patient’s past medical history included traumatic brain injury, seizures, quadriplegia, dysphagia with gastrostomy tube placement, colostomy and colectomy, recurrent polymicrobial infections of the sacral decubitus ulcer, history of pulmonary embolism, chronic atrial fibrillation, urinary retention necessitating indwelling urinary catheterization, and a recent hospitalization in Las Vegas for anemia in June of 2023. During this admission, the patient was started on vancomycin and piperacillin-tazobactam for empiric treatment of suspected chronic sacral osteomyelitis. Cultures obtained prior to surgical debridement grew methicillin-resistant staphylococcal aureus (MRSA) and *Proteus mirabilis*. The debridement was uncomplicated, and the patient was subsequently discharged on oral linezolid with a wound vacuum device, alongside plans for close follow up and wound care management. The wound cultures revealed multiple organisms which included light *C. auris* growth, but was most significant for *Klebsiella*, *Pseudomonas*, MRSA, and *P. mirabilis*. *C. auris* was identified as originating from clade III, with antifungal susceptibility testing indicating resistance to fluconazole, with a MIC of >256 mcg/mL. The MIC to voriconazole was 2 mcg/mL, itraconazole was 1 mcg/mL, posaconazole and isavuconazole were 0.5 mcg/mL, respectively, although interpretative breakpoints are not currently established. The MICs to amphotericin B and anidulafungin were noted to be at 1 mcg/mL, and the MICs to caspofungin and micafungin were 0.5 mcg/mL, respectively, suggesting in vitro susceptibility to these agents.

The patient was admitted to this facility in September 2023 for sepsis with encephalopathy. The patient was found to have *Proteus mirabilis* bacteremia and was subsequently treated with a short antibacterial course including meropenem, tobramycin, and vancomycin, which were later changed to linezolid and cefiderocol. Additionally, the patient was treated with micafungin because of the known history of *C. auris*. One month after discharge, a routine outpatient sacral wound culture grew numerous enteric organisms with no growth of *C. auris*. Subsequent outpatient sacral wound culture grew light growth of ESBL *Klebsiella*, MRSA, and *C. auris*. *C. auris.* MIC data from this culture yielded no increases in resistance with the MIC to fluconazole, noted at >256 mcg/mL, 2 mcg/mL for voriconazole, 0.5 mcg/mL for amphotericin B, 0.25 mcg/mL for anidulafungin, itraconazole, and micafungin, and 0.12 mcg/mL for isavuconazole and posaconazole. During a later hospitalization in April 2024, light growth of *Proteus*, as well as *Actinobacter* were reported on a left hip wound culture. In a subsequent hospital stay in May 2024, a wound culture obtained from the patient’s sacrum grew normal skin flora with no *C. auris* isolated.

The patient was hospitalized in September 2024 and passed away due to septic shock secondary to *Staphylococcus aureus* bacteremia.

### 3.2. Case 2

In June 2022, a 23-year-old male presented to our hospital for a right hip abscess and presacral abscess as well as longstanding sacral osteomyelitis. The patient’s past medical history was significant for the development of a multiloculated abscess in the presacral, right gluteal, and epidural regions, with blood cultures that grew group F streptococcus. Since then, the patient has had chronic lumbar osteomyelitis at the L5 region, recurrent right hip abscess, and presacral abscess with fistula. The patient also had a recent prior hospitalization for an intra-abdominal abscess and appendicitis with perforation. Additionally, the patient had a history of traveling from Reno to Las Vegas as a planned transfer to a hospital in Las Vegas. After returning to our facility for continued management of these abscesses and sacral osteomyelitis, the right hip wound was cultured and demonstrated light *C. auris* growth. After this, the patient was subsequently treated with intravenous micafungin during the duration of his clinical course. The *C. auris* isolate was sequenced and noted to also originate from clade III. Antifungal susceptibility testing revealed resistance to fluconazole with a MIC of >256 mcg/mL. MICs to voriconazole were 4 mcg/mL, 1 mcg/mL for anidulafungin, 0.5 mcg/mL for caspofungin, itraconazole, micafungin, and 0.25 mcg/mL for amphotericin B, posaconazole, and isavuconazole.

The patient was treated with posaconazole in the outpatient setting for continued therapy of *C. auris* infection for a duration of 3 months. No further wound cultures or sensitivities were drawn, and subsequent blood cultures were negative for growth.

In 2024, the patient had multiple admissions to the hospital for intra-abdominal abscesses secondary to perforated appendicitis and later cholecystitis. These were resolved in June 2024 with drainages performed by interventional radiology, and a laparoscopic cholecystectomy. Wound cultures were not obtained, and blood cultures did not show growth of any organism. The patient was discharged home with close planned follow-up, but the patient was subsequently lost to further follow-up.

### 3.3. Genome Sequencing

Genome sequencing performed by the Nevada State Public Health Laboratory revealed that both *C. auris* isolates belonged to clade III with phenotypic resistance to fluconazole. No genetic mutations of concern for antifungal resistance in FKS1, ERG11 or FUR1 were identified in either case. Utilizing a shared core single nucleotide polymorphism (SNP) analysis, a comparison of 83 closely related isolates to the isolates from Patient 1 ranged from being genetically identical to a maximum difference of 8 shared SNPs. Further comparison of 190 closely related isolates to the clinical sample from Patient 2 ranged from genetically identical to a maximum of 5 shared SNPs difference.

## 4. Discussion

*C. auris* remains a significant and growing concern due to high levels of clade-specific antifungal drug resistance including occasional reports of pan-drug resistance. Here, we present two cases of *C. auris* isolated from patients with chronic wounds who had recent travel and hospitalization in a healthcare facility in Las Vegas.

In both cases, only light growth of *C. auris* was demonstrated. Taken together with the rest of the cultures and the clinical scenario, the *C. auris* infections were consistent with mixed polymicrobial infections involving other common healthcare-associated bacterial pathogens. Both cases were treated with micafungin, with Patient 1 never clearing the infection, as demonstrated by recurrent light growth, and Patient 2 not being re-cultured. Patient 2 was subsequently treated with an extended course of posaconazole in the outpatient setting for further therapy. Both patients were otherwise treated conventionally for their abscesses, and did not develop more invasive or disseminated *C. auris* infection. In both cases, it appeared that *C. auris* had a minor role in the overall course of infection, since neither patient developed fungemia nor severe local invasion from *C. auris*.

Data from the Nevada Department of Health and Human Services indicates that within the state of Nevada (as of 13 January 2025), most cases of *C. auris* colonization or clinical infection were noted to be in southern Nevada, particularly Las Vegas. Across the hospitals in northern Nevada, there have been only three confirmed clinical cases and seven additional cases of *C. auris* colonization in the same time period. With the known burden of *C. auris* in healthcare facilities in Las Vegas, the patients’ recent travel to Las Vegas, and the confirmed stay of Patient 2 at an acute care hospital with a *C. auris* outbreak, it is highly likely the patients in this report acquired *C. auris* in southern Nevada. This hypothesis was confirmed using genotyping and clade analysis performed by the Nevada State Public Health Lab, demonstrating direct clonal acquisition and spread from one region of the state to the northern part of the state, a distance of approximately 450 miles. Finally, with the nosocomial nature of *C. auris*, and the confirmed hospital stays for both patients in Las Vegas, it is highly likely that the source of the acquisition was at the Las Vegas healthcare facility.

## 5. Conclusions

In the two cases of *C. auris* in our facility, both were found to belong to clade III and demonstrated resistance to fluconazole. Both cases had recent travel to Las Vegas, and most likely acquired *C. auris* at a healthcare facility there, which was subsequently confirmed through genome sequencing. Facilities at the center of the fungal outbreak and facilities accepting transfers from high-burden areas should be aware of the consequences of fungal spread and ensure appropriate testing for patients upon transfer. A high degree of suspicion and screening of patients should be continued for *C. auris* during the next stages of the geographic spread of *C. auris*. If *C. auris* is identified, isolation precautions should be implemented due to the ease of spread of this nosocomial, opportunistic fungus.

### Strengths and Limitations

Strengths include the ability to see a high proportion of the clinical cases in northern Nevada and the ability to genetically identify the isolates as identical to the clades present in the greater Las Vegas area. Weaknesses include the paucity of total cases and limited follow up for the living patient.

## Data Availability

Genetic data may be made available from the Nevada State Public Health Laboratory upon request to maintain patient privacy. SSR numbers for sequencing data are available in Supplement S1.

## Supplementary Materials

The following supporting information can be downloaded at: https://www.mdpi.com/article/10.3390/jof11060445/s1, Supplement S1: Sample SRR Numbers.

## Abbreviations

The following abbreviations are used in this manuscript:

SNF	Skilled Nursing Facility
MRSA	Methicillin-Resistant Staphylococcus Aureus
SNP	Single Nucleotide Polymorphism
MIC	Minimum Inhibitory Concentration
