# Supplementary material for: Genetic Confirmation of Clonal Spread of Candida auris from Southern to Northern Nevada
Source: J Fungi (Basel). 2025 Jun 12;11(6):445. doi: 10.3390/jof11060445 (PMC12194799; doi:10.3390/jof11060445)
Supplement: Supplementary file 1 [file jof-11-00445-s001.zip › jof-3681725-supplementary.pdf]

Supplement 1: Sample SRR Numbers
These numbers represent the isolate’s DNA sequence which can be accessed through the NIH Library of Medicine

Patient_A_001 - SRR20081642

Patient_B_001 - SRR26968290

Patient_B_002 - SRR30844559
